# Embracing Artificial Intelligence in Dental Practice: An Exploratory Study of Romanian Clinicians’ Perspectives and Experiences

**DOI:** 10.3390/dj13090390

**Published:** 2025-08-27

**Authors:** Alin Flavius Cozmescu, Ana Cernega, Dana Galieta Mincă, Andreea Cristiana Didilescu, Marina Meleșcanu Imre, Alexandra Ripszky Totan, Simona Pârvu, Silviu-Mirel Pițuru

**Affiliations:** 1 Department of Organization, Professional Legislation and Management of the Dental Office, Faculty of Dental Medicine, “Carol Davila” University of Medicine and Pharmacy, 17-23 Plevnei Street, 020021 Bucharest, Romania; alin-flavius.cozmescu@drd.umfcd.ro (A.F.C.); silviu.pituru@umfcd.ro (S.-M.P.); 2 Discipline of Public Health and Management, Faculty of Medicine, “Carol Davila” University of Medicine and Pharmacy, 050474 Bucharest, Romania; 3 Department of Embryology, Faculty of Dentistry, The “Carol Davila” University of Medicine and Pharmacy, 050474 Bucharest, Romania; andreea.didilescu@umfcd.ro; 4 Department of Prosthodontics, Faculty of Dental Medicine, “Carol Davila” University of Medicine and Pharmacy, 17-23 Plevnei Street, 020021 Bucharest, Romania; marina.imre@umfcd.ro; 5 Department of Biochemistry, Faculty of Dental Medicine, “Carol Davila” University of Medicine and Pharmacy, 17-23 Plevnei Street, 020021 Bucharest, Romania; alexandra.ripszky@umfcd.ro; 6 National Institute of Public Health, Faculty of Medicine, “Carol Davila” University of Medicine and Pharmacy, 050474 Bucharest, Romania; simona.parvu@umfcd.ro

**Keywords:** artificial intelligence, doctor–patient relationship, healthcare system, education, management, diagnostics, treatment

## Abstract

**Background/Objectives:** Standard dental practice is being reshaped by digital technologies, and artificial intelligence (AI) is emerging as one of the most challenging recent innovations. **Methods:** The present study assessed the interest of Romanian dentists in the integration of AI into their current practice through an anonymous questionnaire distributed to 200 respondents. The questionnaire addressed the integration of AI in dentistry by analyzing the following areas of intervention: stages of patient care, perceived impact on the doctor–patient relationship, data security, implementation costs, and the legislative framework. **Results:** The results showed that 53.6% of dentists reported low difficulties, 37.3% reported moderate difficulties, and 9.1% reported high difficulties with using digital tools. Dentists’ reported willingness to adopt AI-based solutions was as follows: 58.6% were very willing, 30% were moderately willing, and only 11.4% were not very willing. Currently, 80.5% already use digital techniques in their daily practice. The participants emphasized the need to maintain a strong doctor–patient relationship while recognizing the benefits of increased efficiency. They were aware of the risk of diminishing human connection and trust. Also, data security and the financial stress associated with implementing and maintaining new systems were seen as major obstacles. **Conclusions:** The dentists surveyed showed an increased interest in modern digital technologies, provided that there is a clear legal framework, a strong data protection system, and the preservation of the doctor–patient relationship based on trust and confidentiality that defines the medical profession.

## 1. Introduction

### 1.1. A Brief History of Artificial Intelligence and Its Applications in Dentistry

Artificial intelligence (AI), in its modern sense, refers to computer technologies that are capable of mimicking human cognitive functions such as learning, decision-making, and complex reasoning. Alan Turing, a key pioneer of artificial intelligence, proposed the “Turing Test” in 1950 to judge whether a machine can imitate human behavior convincingly enough to deceive an interrogator communicating solely through written messages. If the interrogator cannot reliably distinguish the machine from a human participant, the machine is considered to have demonstrated human-like intelligence [1,2].

In 1956, mathematician John McCarthy, who is considered the father of AI, introduced the term at a conference held in Dartmouth, USA, officially marking AI as an independent field of research [3]. Recent decades have seen significant advancements in machine learning algorithms and deep neural networks, enabling AI to perform tasks with an accuracy comparable to human experts and particularly transforming the healthcare sector. Dentistry has been among the early adopters of AI, employing AI-driven tools for precise diagnosis and personalized treatment planning [4]. Deep learning algorithms now allow analysis of dental radiographs and intraoral images to detect caries, periapical lesions, periodontal diseases, and early-stage malignancies [5]. Additionally, AI has improved implantology by accurately predicting implant osseointegration, planning prosthetic restorations, and identifying specific implant types, thus saving substantial time and resources. These decision-support systems also reduce diagnostic variability, standardize clinical procedures, and improve patient communication [6,7]. Although AI-based applications that can present patients with the anticipated outcomes of orthodontic treatment may, in the future, become valuable tools for supporting therapeutic decision-making, according to a study by Tomášik et al., their current utility remains limited due to technical, regulatory, and ethical constraints. Consequently, physician–patient dialogue and individualized treatment plans remain indispensable [8].

To outline a clearer and more comprehensive picture regarding the future of AI in the healthcare sector, we conducted a multidimensional study based on the three primary components of the dental healthcare system: the dentist, who represents the core element of expertise and clinical practice; the dental practice manager, who is responsible for the administrative and organizational dimension of the system; and finally, the patient, who is the direct beneficiary of the development and improvement of therapeutic options [9,10]. We chose the perception of dentists towards the integration of AI into current clinical practice as our initial focus and research starting point, as the dentist constitutes the fundamental actor of the dental healthcare system [11]. Rooted within the doctor–patient relationship, the dentist acts as the central figure in the structure of medical practice, accountable not only for the success of the therapeutic plan but also for preserving the human dimension of the caregiving process [12]. The dentist remains the cornerstone of the interpersonal relationship with the patient, which is an essential aspect of the success of every treatment plan. Empathy, communication, and trust are core shared values that underpin this relationship. Moreover, the dentist–patient interaction can be viewed as a microsystem within dental practice [13,14]. This microsystem consists of five successive stages: patient scheduling, diagnosis, treatment planning and implementation, patient follow-up (dispensarization), and post-treatment feedback. Each of these five stages involves specific interactions which will form the basis of our research, as these are common steps performed by every dentist in clinical practice. Each component and stage also represent individual pieces of a broader puzzle that forms the overall healthcare system [15]. This macro-system is witnessing a significant paradigm shift—the emergence and integration of AI in every phase of dental treatment [16]. Without clear and precise regulation regarding the implementation of these new technologies, the doctor–patient relationship risks experiencing disruptive changes, potentially damaging and altering the relationships built over years of clinical practice. In order to obtain an overall view of the previously described systems and actors, we created a diagram to illustrate the roles, characteristics, and underlying mechanisms of the dental healthcare system. It also highlights the benefits and vulnerabilities arising from the implementation of AI at various levels and stages—managerial, clinical, and patient-level. These benefits and risks emerged from a review of the specialized literature and paved the way for further discussions regarding the individual role this technology plays in enhancing the medical act and, ultimately, in promoting patient-related health and satisfaction [17].

### 1.2. Legislative Context

Given that AI is a field with a pronounced dynamic, the legislative spectrum plays an extremely important role in its regulation [18]. The European Union, through the General Data Protection Regulation [19] and the EU Artificial Intelligence Act [20], comes to the aid of all actors involved in this process through regulations designed to protect both the interests of the doctor and the patient from possible abuses or legislative gaps. As medical information represents one of the most valuable information resources and has a high degree of protection, health professionals play a key role in obtaining, using, and protecting this information [20].

The transition to an increasingly digital world in which AI makes its presence felt more and more, as well as the paradigm shift and the increased agency of the patient within the doctor–patient relationship, represents a historical moment, comparable to the moment of the appearance of the Hippocratic Oath.

The study presented in this scientific article is part of a broader research initiative designed to answer three fundamental questions concerning dentists’ perceptions of AI integration into clinical practice: What level of difficulty do dentists experience in utilizing AI within dental practices? How willing are dentists to adopt new technologies based on AI? Lastly, what is the impact of AI-driven digital tools on their current clinical practices?

Beyond the perspectives of dental professionals, this research project also aims to explore insights from dental practice managers, representing the administrative and organizational dimensions of the broader dental healthcare system, as well as patient perspectives on these transformative changes. These additional viewpoints will be discussed in forthcoming scientific articles, ultimately assembling a comprehensive and multidimensional picture of this evolving landscape.

## 2. Materials and Methods

### 2.1. Statistical Methods

The Materials and Methods section outlines the research design, participant selection, data collection instruments, procedural steps, and analytical techniques employed in this study. In conducting this study, we applied the STROBE recommendations required for cross-sectional studies, thus ensuring a high degree of rigor in the work [21]. A quantitative approach was adopted to investigate dental practitioners’ perceptions and attitudes toward digitalization and AI in clinical practice.

Statistical analysis was performed using IBM SPSS Statistics for Windows, version 25 (IBM Corp., Armonk, NY, USA) and Microsoft Office Excel/Word 2024. Quantitative variables were reported as means with standard deviations or medians with interpercentile ranges; their distribution was assessed using the Shapiro–Wilk test. Non-parametric independent quantitative variables were compared between investigated factors via Kruskal–Wallis H tests (with Dunn–Bonferroni post-hoc tests), while correlations between non-parametric independent quantitative variables were evaluated using Spearman’s rho correlation coefficient. Qualitative variables were expressed as absolute values or percentages, and intergroup differences were tested with Fisher’s Exact Test. Z-tests with Bonferroni corrections provided further detail in contingency tables.

The investigated subdomains were as follows: scheduling, diagnosis, treatment planning, feedback, and follow-up (dispensarization), each containing 15 items in the survey. In each of the subdomains, exploratory Principal Component Analysis (PCA) models were used, retaining the first component with the highest explained variance (largest eigenvalue, λ), and with items included in the first component having at least an absolute loading of ≥0.30. Analyzing all items in the survey, an overall exploratory PCA was made in order to observe overall digitalization scores, in which four components emerged.

Each item received a score from 1 (minimum interest) to 5 (maximum interest), reversed for items showing negative PCA coefficients. The overall score was the arithmetic mean of all included items. PCA was used only for the exploration of item loading patterns, in which items that needed reverse scoring could be identified; as such, validation assessments for PCA models were not necessary.

Observed scores in the exploratory PCA models were validated via Cronbach’s alpha, where internal consistency was measured, examining changes in the coefficient upon item removal. Statistical significance was set at α = 0.05.

### 2.2. The Design of the Questionnaire

Data collection was carried out through an anonymous structured questionnaire administered on paper (Supplementary Materials). The questionnaire was developed de novo to align with the recent evolution of AI in healthcare and the changing dynamics of the dentist–patient relationship. Existing validated instruments did not adequately capture these emerging aspects; therefore, we designed the items specifically for this study to ensure contextual relevance. Furthermore, the questionnaire was adapted to the needs and comprehension style of Romanian dental practitioners, taking into account the specific workflow characteristics in Romania.

Three dependent variables were evaluated: perceived difficulty in utilizing digital tools, willingness to integrate AI technologies, and perceived impact of AI on routine clinical practice. The questions were designed to analyze the five key stages of a patient’s visit to a dental clinic: scheduling appointments, diagnosing dental conditions, planning treatment, patient feedback, and follow-up/dispensing procedures. The variable regarding perceived difficulty was identified from a set of questions that sought to identify possible challenges that may arise in the five stages mentioned above (efficient time management, compliance with legislation, operational efficiency, communication, and compliance with internal regulations). The variable regarding willingness to adopt AI-based technologies was measured through a parallel set of questions focused on identifying and classifying opportunities for improvement through digital technologies (optimization of medical times and processes, integration and interoperability of systems, preliminary and personalized assessment, extended access to users, and updating and simplifying information). The perceived impact was also assessed by analyzing responses to questions about the potential threats posed by the integration of digital processes and artificial intelligence (technological obsolescence, data security risks, algorithm errors, excessive dependence on automation, and depersonalization of communication).

### 2.3. The Studied Group

As the main eligibility criterion, the selection was made among clinicians actively involved in dental practice in Bucharest and adjacent areas who were willing to complete and participate in this research. In order to qualify, participants had to hold one of the following professional qualifications officially conferred by the Romanian dental regulatory authority: general dentist, resident, specialist, or primary care physician. There were no restrictions on age, gender, or seniority in clinical practice. Practitioners who did not routinely provide direct dental care to patients were excluded.

As a method of selection, participants were recruited through purposive and convenience sampling methods, employing direct communication via professional channels and online platforms frequently accessed by Romanian dental professionals.

The study population comprised dental professionals across different career stages, including general dentists, residents, specialists, and senior practitioners. Independent variables encompassed demographic and professional attributes, specifically age, gender, practice setting, professional status, and clinical experience level. This combined methodological framework aimed to yield a nuanced understanding of factors influencing the adoption of AI and digital technologies within dentistry.

To ensure methodological rigor, these demographic and professional categories were determined through a structured questionnaire administered under standardized conditions. Informed consent was obtained from all participants prior to data collection, and responses were aggregated and analyzed in accordance with established ethical and academic standards. These stratifications and demographic summaries provided an essential foundation for interpreting this study’s subsequent findings. The questionnaire was reviewed and approved by the Scientific Research Ethics Committee of the Carol Davila University of Medicine and Pharmacy, Bucharest. This approval bears the code PO-35-F-03 and has the registration number 28,287 from 1 October 2024.

This study was conducted over a period spanning from October 2024 to March 2025. No missing data were present, as all returned questionnaires were fully completed.

No formal sample size calculation was performed, as this research was designed as a pilot study to address AI interference in the doctor–patient relationship. Given the early stage of the study, we identified a number of limitations in its implementation, such as the different distribution of physician categories according to age (with younger physicians being more open to completing the survey), their experience, professional level, work environment (urban and rural), and the time when the research was conducted. These limitations can be addressed in future research. The current study represents a starting point and orientation for future approaches.

## 3. Results and Discussions

### 3.1. Demographic Results

The findings of this study emphasize commonly held views on the acceptance and use of AI-driven digital technologies while also revealing issues that warrant further scrutiny. This Results section offers an overview of dentists’ attitudes, highlighting both established practices and unresolved questions surrounding the integration of AI into clinical workflows. In total, 200 dentists completed the questionnaire, providing data on their attitudes toward digitalization and the integration of AI in dental practice, perceptions about doctor–patient relationships, data security concerns, implementation costs, and legal implications. The study sample consisted of dental practitioners selected from a total population of 5708 dentists officially registered with the College of Dentists in Bucharest [22]. Participants were recruited from dental practices situated in Bucharest and its surrounding areas, including adjacent rural communities.

#### 3.1.1. Distribution of Participants by Age

Within the framework of this study, the participants were stratified into specific age groups with the following distribution: 25.9% aged 25–30 years, 30% aged 31–35 years, 16.4% aged 36–40 years, 11.8% aged 41–45 years, 7.7% aged 46–50 years, 3.2% aged 51–55 years, 4.5% aged 56–60 years, and 0.5% aged over 60 years. In terms of gender representation, 57.7% of respondents were male, while 42.3% were female. Regarding the primary practice setting, the majority (85.9%) of participants reported working in urban areas, with 14.1% working within rural environments.

#### 3.1.2. Distribution of Participants by Professional Qualification

Conducted in Romania—where dental school graduates may hold one of the following professional qualifications: general dentist, resident, specialist, or primary dentist—this study found that 55.9% of respondents were general dentists, followed by specialists (29.5%), residents (9.5%), and primary dentists (5%). Participants were further categorized based on years of clinical experience: 26.4% had 0–3 years, 36.8% had 4–10 years, 17.7% had 11–15 years, 10% had 16–20 years, 3.6% had 21–25 years, 4.5% had 26–30 years, and 0.9% had more than 31 years of experience.

In order to gain a more nuanced understanding of how willing dental practitioners are to transition toward an increasingly digital working environment and to incorporate applications and technologies supported by various AI systems into their routine practice, the questionnaire included three specific items aimed at distinguishing participants’ competencies, readiness, and current stage of digital technology adoption.

### 3.2. Subdomains of Dental Treatment

Stages such as scheduling, establishing a diagnosis, treatment planning, dispensing, and feedback are the focus of current and future digital tools. Maintaining the doctor–patient relationship, efficiency, data interoperability, and concerns regarding data security and protection represented key elements in the concerns of dentists throughout the questionnaire.

**Figure 1:**
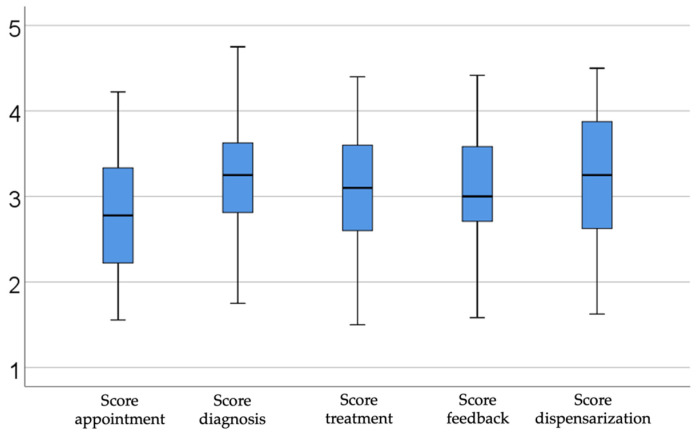
Illustrating scores by subdomains.

Figure 1 provides an overview of the interest scores for five key subdomains—scheduling, diagnosis, treatment planning, feedback, and follow-up (dispensarization)—in the dental care process. The highest scores were observed for diagnosis (median = 3.25, IQR = 2.78–3.62) and follow-up (median = 3.25, IQR = 2.62–3.87), indicating moderate-to-high levels of interest. Diagnosis involves the identification of oral conditions and the formulation of clinical judgments, while follow-up focuses on ongoing care, preventive measures, and the monitoring of treatment outcomes. Treatment planning (median = 3.100, IQR = 2.60–3.60)—which translates diagnostic findings into a structured intervention strategy—received moderate interest, suggesting recognition of its importance without it dominating overall attention. Feedback, reflecting the post-treatment exchange of information between dentist and patient, also showed moderate interest, emphasizing the value of patient satisfaction and continuous improvement. By contrast, scheduling yielded the lowest score (median = 2.77, IQR = 2.22–3.33), with only moderate-to-low concern regarding the administrative aspects of appointment-setting.

The results of this analysis highlight clear trends regarding dentists’ interest in integrating AI at different stages of clinical activity. The analysis of the subdomains relevant to dental practice, namely patient scheduling, diagnosis, treatment planning, feedback, and dispensing, reflects varying levels of concern and readiness for the adoption of innovative technologies based on AI tools.

#### 3.2.1. Analysis of Subdomain—Diagnosis

Diagnosis, an essential stage involving the identification of various oro–maxillo–facial conditions with resonance and effects on the health of the whole organism and the formulation of coherent clinical reasoning, saw high interest, suggesting that dentists perceive AI as a valuable resource in the accuracy and speed of clinical decisions. This trend indicates that dentists recognize the potential of technology in supporting their own expertise and reducing diagnostic errors, as well as in improving the quality of patient care. A comparable interest was also identified in the patient’s dispensary stage, which includes monitoring treatment results, prevention, education, and ongoing care. This result shows that doctors understand the importance of using digital technologies for the long-term follow-up of oral health and the early identification of possible complications, essential aspects of the doctor–patient relationship and loyalty. These solutions can improve patients’ awareness of oral health and stimulate the adoption of effective preventive behaviors. Thus, the use of technology for education and prevention can translate into reducing the incidence of oral diseases, optimizing long-term therapeutic costs, and strengthening the doctor–patient relationship by actively involving patients in managing their own oral health. This approach reflects a fundamental paradigm shift from predominantly curative treatment to preventive dentistry and is oriented towards continuous patient education, optimally using digital tools and AI as essential support in this process.

#### 3.2.2. Analysis of Subdomain—Treatment Planning

Treatment planning, which involves structuring interventions based on diagnostic information, saw moderate interest, which reflects an awareness of the relevance of this stage, but also a relative reluctance to fully adopt new digital tools. It is crucial to note that one potential reason may be the difficulty of integrating cutting-edge technical solutions into everyday operations; this is an area that needs more focus through specialized training courses and user-friendly interfaces.

#### 3.2.3. Analysis of Subdomain—Feedback

When discussing the interest expressed regarding feedback after completing dental treatments, which involves a broader discussion with the patient regarding the results expected by them and the actual results of the treatment, the response of the doctors and their degree of interest was moderate. The doctors were aware of the importance of better communication with the patient and the degree of satisfaction and trust felt by the patient. Still, they were not fully prepared to adopt a completely digital or mostly digital approach to this stage. To improve this aspect, it is essential to develop systems that facilitate the rapid assessment of patient satisfaction, integrating intuitive and easily accessible digital technologies.

#### 3.2.4. Analysis of Subdomain—Appointment

On the other hand, the lowest interest was observed in the appointment stage, which suggests that dentists consider managing appointments as a less relevant task for their direct clinical work. This attitude may emphasize the perception that administrative activities are secondary compared to actual medical decisions. From another perspective, this lack of focus on using AI in the programming phase can be justified by doctors’ desire to maintain a human touch in administrative processes, so that they can respond to patients’ needs for safety through human connection. However, increasing interest and adoption of digital technologies in this stage can optimize the workflow, reduce time lost in administrative activities, and allow for better focus on clinical aspects and direct patient relationships.

Overall, these results underscore the priority placed on direct clinical decision-making and continuous patient engagement over preliminary logistical tasks.

### 3.3. The Perceived Degree of Difficulty

To create a more accurate understanding of the interviewed dental practitioners’ perspectives, they responded to a question evaluating the degree of difficulty encountered when using digital tools within their current practice. The findings from our study highlight an improved perception among dental practitioners regarding the level of difficulty experienced when utilizing digital instruments. Specifically, only 9.1% of respondents indicated a high degree of difficulty, 37.3% reported a moderate degree of difficulty, and 53.6% perceived a low degree of difficulty. A detailed analysis according to age categories, professional experience, and practice setting yielded the following results.

#### 3.3.1. Analysis of Perceived Difficulty by Age Group

Table 1 depicts how dentists of different ages experience varying levels of difficulty with digital tools and information technology. According to Fisher’s Exact Test (*p* < 0.001), these differences are statistically significant. Post-hoc Z-tests with Bonferroni corrections indicate that dentists aged 31–35 years reported low difficulty most often (40.7%, compared to 20.7% and 5% in other groups). By contrast, those aged 46–50 (20% vs. 3.4%), 56–60 (15% vs. 1.7%), and over 60 (5% vs. 0%) more frequently reported a high degree of difficulty. Additionally, dentists aged 51–55 tended to report moderate or high difficulty (15%/4.9%) rather than low difficulty (0%).

#### 3.3.2. Analysis of Perceived Difficulty in Relation to Professional Experience

Table 2 presents the relationship between dentists’ professional experience (years in practice) and their reported difficulty in utilizing digital instruments. Statistical analysis using Fisher’s Exact Test revealed significant differences among groups (*p* < 0.001). Subsequent post-hoc analyses employing Z-tests with Bonferroni corrections indicated that dentists with 4–10 years of professional experience were significantly more likely to report low difficulty rather than moderate or high difficulty (48.3% vs. 28%/5%). Conversely, practitioners with greater experience—specifically those within the intervals of 21–25 years (15% vs. 0.8%), 26–30 years (20% vs. 1.7%), and 31–35 years (5% vs. 0%)—reported high difficulty significantly more frequently than low difficulty.

The data underscore a clear correlation between professional experience and perceived difficulty with digital tools, indicating that younger dentists, or those with fewer years in practice, tend to adapt more readily to digital technologies. Conversely, practitioners with extensive professional experience may face greater challenges, highlighting the importance of further discussions and studies to bridge gaps among more experienced professionals.

#### 3.3.3. Analysis of the Perception of the Degree of Difficulty in Relation to the Professional Environment

**Figure 2:**
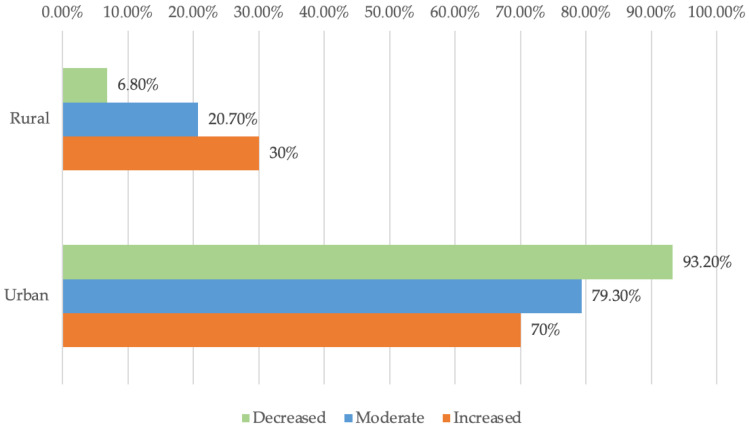
Degree of difficulty—urban vs. rural.

The data presented in Figure 2 illustrate the distribution of dental practitioners based on practice setting and perceived degree of difficulty. Differences between groups, evaluated through Fisher’s Exact Test, were statistically significant (*p* = 0.001).

Furthermore, Z-tests with Bonferroni correction indicated that rural practitioners reported a moderate or high level of difficulty rather than a low level (30%/20.7% vs. 6.8%) significantly more frequently, whereas urban practitioners reported a low level of difficulty rather than moderate or high levels of difficulty (93.2% vs. 79.3%/70%) significantly more frequently.

These findings highlight a significant variation in perceived difficulty concerning the use of digital instruments between urban and rural dental practitioners. Specifically, rural dentists reported encountering higher levels of difficulty significantly more often than their urban counterparts. Several factors may explain this discrepancy. While infrastructure challenges remain significant, individual barriers—such as digital literacy and limited peer support—often hinder rural dentists’ adoption of new technologies. Viewing this gap as an opportunity for targeted empowerment at the practitioner and clinic level can make a substantial impact.

### 3.4. The Degree of Willingness

#### 3.4.1. Analysis of Willingness by Age Group

Table 3 illustrates how different age groups vary in their willingness to adopt new AI-based technologies in dental practice. Fisher’s Exact Test indicates a statistically significant difference between groups (*p* < 0.001). Post-hoc Z-tests with Bonferroni corrections show that dentists aged 31–35 years were more likely to display a high level of willingness compared to a moderate level (38.8% vs. 18.2%), whereas those aged 41–45 more often reported a moderate level of willingness rather than a high one (19.7% vs. 7.8%).

When examining the willingness of dental practitioners to adopt new, AI-based technologies, psychological and behavioral factors play a pivotal role. The way a dentist perceives and approaches technology adoption is closely tied to their interpretation of the new information made available to them. Professional socialization further contributes to this attitude. Younger dentists have been socialized in an era of rapid technological advancement and likely experienced mentors or curricula encouraging the use of digital tools. Meanwhile, many older dentists were socialized under earlier norms that emphasized manual expertise and physician autonomy, which might predispose them to view AI as an external disruptor rather than an integral part of care. Risk perception is another differentiator: older clinicians are generally more risk-averse, which can lead them to perceive implementing AI as a potential risk, whether due to technical uncertainties, patient safety concerns, or disruptions to workflow, that they are reluctant to undertake this new technology without clear proof of benefit. Younger clinicians, having slightly lower risk aversion on average and fewer ingrained practice habits, may be more willing to pilot new technologies with the hope of gaining efficiency or improved outcomes. Lastly, perceived threats to professional autonomy and role identity influence adoption. Some experienced dentists fear that AI could encroach on their decision-making authority or undermine their expertise, fueling reluctance to adopt tools that might dictate or second-guess their clinical judgment. In contrast, the younger clinicians accustomed to working with decision-support software and evidence-based guidelines, may tend to see AI as an augmentative partner that streamlines workflows rather than a threat to their autonomy. These sociocultural and psychological factors together help explain why the younger group is more enthusiastic about integrating AI, and they suggest that as tech-confident generations become the majority, the overall trajectory of AI adoption in dental practice will accelerate, driven by those younger professionals who are shaping the future and setting new trends in the field.

#### 3.4.2. Analysis of Willingness in Relation to Professional Experience

Table 4 illustrates how dentists’ years of experience correlate with their willingness to adopt new technologies. Fisher’s Exact Test confirms a statistically significant difference between groups (*p* < 0.001). Post-hoc Z-tests with Bonferroni corrections indicate that those with 4–10 years of experience more frequently reported high rather than moderate willingness (45.7% vs. 24.2%), while dentists with 11–15 years of experience more often showed moderate rather than high willingness (28.8% vs. 12.4%). Additionally, practitioners with 16–20 years of experience were more likely to report low or moderate willingness instead of high (32%/15.2% vs. 3.1%).

The data also show a dynamic interaction between professional experience and technology acceptance among dentists, suggesting that those with fewer years of practice may be more receptive to innovations. In contrast, those with more experience may be wary of new tools, possibly due to well-established traditional clinical experience and perceived complexities. Correcting these divergences can create a unified practice environment in which all specialists capitalize on technological advances, leading to direct patient benefit and optimizing procedures for the real benefit of the physician by optimizing allocated resources, be they time, financial, or human resources involved. Encouraging peer mentoring to share experiences, both traditional and digital clinical, can benefit both parties involved in this exchange of experiences. Providing easily accessible training resources and highlighting tangible clinical benefits can help align the degree of technology adoption across different levels of experience.

Younger practitioners seem more enthusiastic about adopting new technologies, perhaps due to greater familiarity with digital innovations or more recent training. In contrast, the moderate willingness observed in certain intermediate-age segments suggests a cautious approach, possibly influenced by existing workflows or implementation challenges. It is also important to remember the reasons for some degree of reluctance in terms of the availability, degree of use, and adoption of digital tools. Medical professionals are aware of the security risks that come with collecting large packages of sensitive data, such as medical data, the level of protection that must be offered to these types of data, the costs involved in digitization, integration of artificial intelligence as well as protection solutions, and perhaps most importantly, preserving the doctor–patient relationship.

#### 3.4.3. Analysis of Willingness in Relation to the Professional Environment

**Figure 3:**
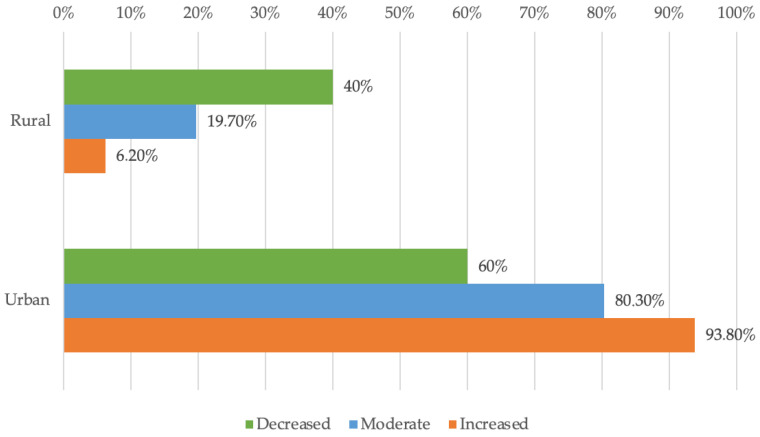
Distribution of doctors according to environment and availability.

Figure 3 compares urban and rural dentists with respect to their willingness to adopt new technologies. According to Fisher’s Exact Test (*p* < 0.001), the differences between groups are statistically significant. Post-hoc Z-tests with Bonferroni corrections show that rural dentists more frequently reported low or moderate willingness rather than high willingness (40%/19.7% vs. 6.2%), while urban dentists more often reported high willingness compared to moderate or low willingness (93.8% vs. 80.3%/60%).

The area of origin of the people who participated in this study highlighted a pronounced digital gap between dentists from urban areas and those from rural areas, reflected by the significant difference in the difficulty of using digital tools. The data suggest that rural dentists show less enthusiasm for adopting new digital solutions than their urban counterparts, who declare themselves more prepared for technological advances and the integration of technologies based on artificial intelligence. With limited access to technological resources and training opportunities that are more difficult to access than their urban colleagues, specialists from rural areas more often reported moderate and high levels of difficulty. The survey results that we managed to obtain during this study suggest an excellent opportunity for training programs and an excellent timeframe to improve technological and digital infrastructure in certain areas, ensuring equal access to the newest digital innovations. The implications include increased competitiveness and higher quality of medical services through the adoption of modern technologies, as well as the need for collaboration between authorities, professionals, and professional organizations to reduce discrepancies. For better results, it is necessary to bridge the gap, ensuring equitable patient experiences, improved diagnostic accuracy, and better workflows across practice types. In connection with the point previously mentioned, investments in continuing distance education can bring a seamless integration of digital technologies into dental practice. This step is crucial for major progress in dental services across the country.

### 3.5. The Use of Digital Tools in Current Practice

#### 3.5.1. Analysis of the Use of Digital Tools by Age Group

Table 5 illustrates how dentists of varying ages differ in their use of digital tools intended to facilitate relationships with patients, staff, and collaborators (e.g., scheduling software, data transfer platforms, and e-learning solutions). According to Fisher’s Exact Test, these differences are statistically significant (*p* = 0.003). Post-hoc Bonferroni-adjusted Z-tests indicate that dentists aged 31–35 years reported using digital tools more frequently (33.3% vs. 16.3%), whereas those aged 45–46 years were more likely to report not using them (23.3% vs. 4%).

According to this study’s results, dentists aged 31 to 35 encounter the fewest difficulties in using digital tools, indicating an easier adoption of new technologies. In contrast, more experienced practitioners face more significant challenges, particularly those over 46. We observed a special category of respondents, aged between 25 and 30, who presented some difficulties when using digital tools. These findings highlight a generational gap in digital skills, highlighting the need for well-structured training and support programs addressed to a well-defined target audience. Programs should be designed with the primary intention to help dentists with extensive clinical experience integrate new tools and young and very young dentists who are at the beginning of their careers adopt a fully digital approach. Overcoming these technological hurdles could increase office efficiency, improve patient care, and encourage broader acceptance of innovations.

#### 3.5.2. Analysis of the Use of Digital Tools in Relation to Professional Experience

The data in Table 6 illustrate the distribution of practitioners according to their level of experience and use of digital technologies. The differences observed between groups, based on Fisher’s Exact Test, were statistically significant (*p* = 0.006). Bonferroni-corrected Z-tests indicated that dentists with 4–10 years of experience were more likely to report using digital tools (41.2% vs. 18.6%), whereas those with 16–20 years of experience reported not using them more frequently (23.3% vs. 6.8%).

If we discuss the level of professional experience, the results obtained in the questionnaire indicate a clear correlation between the number of years of professional practice and the perceived difficulty in using digital tools. Mid-career dentists seem more familiar with technology, while more experienced medical professionals often face greater challenges, usually due to work habits formed over time and a lack of contact with recent innovations. The differences observed both from the individual perspective of the respondents and from the results considered as a whole emphasize the need for personalized training programs both at the individual and group levels. These training programs and the allocation of resources adapted to different levels of knowledge and experience aim to strengthen the variety of digital skills of dentists for a modern practice within the office. From a practical perspective, the development and practice of these skills can transform administrative tasks, which are generally seen as time-consuming, into activities that are performed automatically and, at the same time, can improve communication with patients, strengthening the doctor–patient relationship. Overall, the efficiency of clinical activity can increase, and the time allocated to the patient can also increase considerably. Ultimately, reducing the digital skills gap is essential to maintain the pace of growth, innovation, and quality in modern dentistry.

#### 3.5.3. Analysis of the Use of Digital Tools in Relation to the Professional Environment

**Figure 4:**
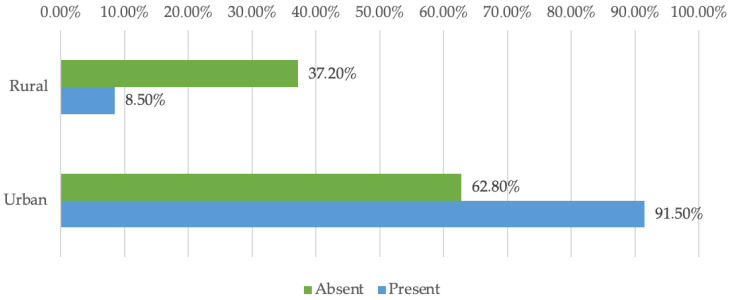
Distribution of doctors by environment and use of digitalization.

Figure 4 illustrates how dentists from urban and rural areas differ in their use of digital tools. Fisher’s Exact Test confirms that these differences are statistically significant (*p* < 0.001). Specifically, urban dentists reported using digital instruments significantly more often (91.5%) compared to those in rural settings (62.8%).

Digitalization and the integration of artificial intelligence allow dentists to overcome the barriers of space, time, and resources. Thus, they facilitate equal and continuous access to specialized services and reduce the geographical discrepancies between urban and rural dental practices. These aspects highlight the need for personalized and differentiated approaches in continuous professional development designed to adapt to the specifics of each targeted professional segment. The information obtained also draws attention to the implementation of personalized support policies and a technological infrastructure specifically adapted to the work environment. These measures are intended to narrow the technology gap and alleviate the identified difficulties. At the same time, they facilitate the acceptance and actual integration of digital techniques and artificial intelligence in dental offices. A more uniform and equitable distribution of dental services can be achieved, which ultimately benefits the patient directly.

### 3.6. Time Management Thinking

**Figure 5:**
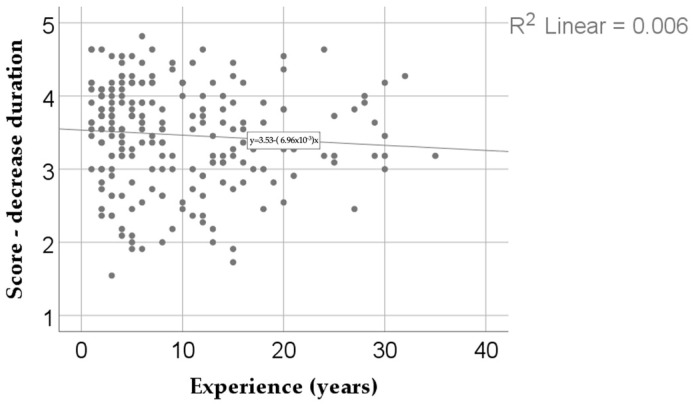
The correlation between the desire score for a decrease in the duration of the medical act and experience.

The data in Figure 5 depict the correlation between the desire-to-reduce-procedure-duration score and experience. Both variables had a non-parametric distribution according to the Shapiro–Wilk test (*p* < 0.05). The observed correlation was significant and negative, albeit of low strength (*p* = 0.044, R = −0.136), indicating that dentists with fewer years of experience were significantly more likely to report a higher desire to shorten clinical procedure time through digitalization, and vice versa.

The results of this study highlight a negative correlation of low intensity between age and the desire to reduce the duration of the medical act through digitization and automation of processes. In other words, younger or less experienced doctors tend to give greater importance to time efficiency, while more experienced practitioners may focus less on this dimension. The fact that the distribution of both variables is non-parametric, confirmed by the Shapiro–Wilk test, indicates a significant heterogeneity of the responses. Although the level of statistical significance validates the relationship, the low strength of the correlation signals that the differences are not uniform and that there are exceptions. Thus, although the general trend suggests that less experienced doctors place greater emphasis on reducing clinical time, some senior practitioners may share the same views, depending on personal factors or the professional context.

Analyzing the results obtained, we can assert that this study identified one of the major perceived benefits of implementing artificial intelligence in the medical sector: time efficiency. The time saved through AI integration does not merely refer to a reduction in the duration of patient interactions but rather represents a comprehensive gain for the entire medical team. Each instance of time optimization across the various stages of treatment constitutes a significant advantage for clinicians, patients, and administrative personnel alike. The relevance of this finding is therefore critical. It can be reasonably argued that artificial intelligence not only contributes to time savings but also plays a role in minimizing errors—particularly those resulting from fatigue, inattention, or burnout. Considering the increasing prevalence of burnout syndrome among healthcare professionals in current practice, AI has the potential to play a decisive role in mitigating its impact and supporting clinical efficiency and well-being.

As a whole, this result confirms the general desire for the efficiency of the medical act and a much more intelligent allocation of one of the most valuable, if not the most valuable, resources: ***time***.

### 3.7. Interest Scores in Relation to Digitalization

**Figure 6:**
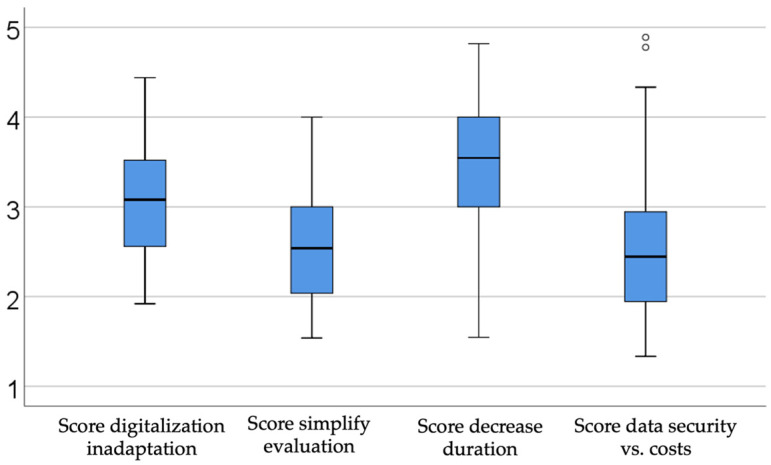
Description of interest scores in relation to digitalization.

Figure 6 summarizes four digitalization-related interest scores among dentists. A moderate level of inadaptation (median = 3.08, IQR = 2.56–3.52) reflects practitioners’ concern for preserving human involvement and skepticism toward fully automating medical processes. A moderate-to-low drive for simplification (median = 2.53, IQR = 2.01–3.00) indicates limited enthusiasm for streamlining evaluations through digital means. In contrast, a moderate-to-high desire to reduce the procedural time (median = 3.54, IQR = 3.00–4.00) suggests a strong interest in efficiency gains. Lastly, a moderate-to-low focus on data security over cost (median = 2.44, IQR = 1.91–2.97) underscores financial considerations when weighing patient-data protection systems. These findings highlight varying degrees of acceptance and caution regarding the integration of digital tools into dental practice.

The results of this study highlight broad trends in digital adoption and usage, identify persistent challenges, and reveal prevailing attitudes toward AI-based technologies. Across sectors, the adoption of digital tools is expanding, yet participants face ongoing challenges in effective implementation and integration. Attitudes toward AI remain generally positive but cautious, reflecting enthusiasm for innovation balanced by awareness of potential risks. This overview of key findings provides a foundation for further analysis, bridging the results to future, deeper exploration.

By analyzing the previously obtained data, a number of advantages can be substantiated for both dental practitioners and their patients. One of the most prominent benefits lies in the reduction in time associated with the digitalization of various medical processes. This reduction shortens the duration of clinical encounters, allowing both the dentist and the patient to gain valuable time. As a result, the doctor–patient relationship is strengthened, and clinical appointments become more efficient and purposeful.

Furthermore, the minimization of potential errors during treatment increases the success rate of implementing therapeutic plans, ultimately leading to higher patient satisfaction and deeper trust between the two parties. The security of medical data plays a crucial role in the process of collecting, using, and storing sensitive health information. When clinicians demonstrate confidence in the technologies employed, this assurance is naturally conveyed to the patient as well. In the context of healthcare, data management is bound by both professional confidentiality and legal obligations related to the protection of personal data.

Ultimately, if these technological implementations are achieved at a cost that both the practitioner and the patient are willing to assume, artificial intelligence can be considered to have fulfilled its objective: to support the development and enhancement of the doctor–patient relationship, along with all its derived dimensions.

### 3.8. Inadaptation to Digitalization

**Figure 7:**
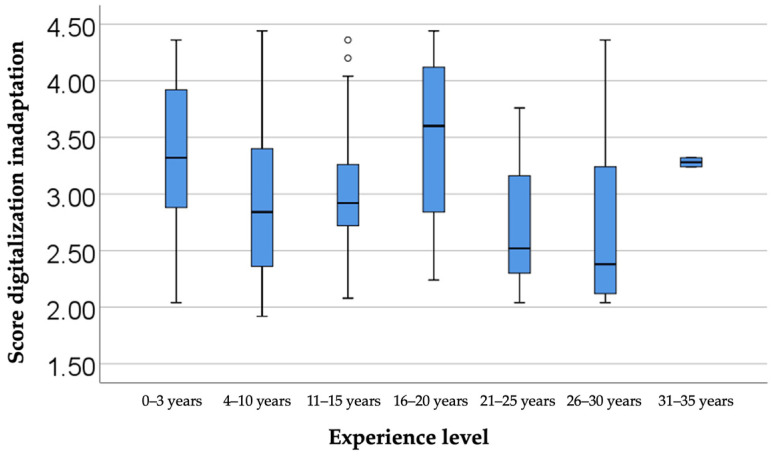
A comparison of maladaptation scores in relation to the level of experience.

The last item that we discussed is presented in Figure 7, which compares the inadaptation to digitalization score according to years of professional experience. The differences observed among the groups, as indicated by the Kruskal–Wallis H test, were significant (*p* < 0.001). Moreover, the Dunn–Bonferroni post-hoc tests revealed that physicians with 4–10 years of experience had a significantly lower maladaptation score (median = 2.84, IQR = 2.36–3.4) compared to those with 0–3 years of experience (median = 3.32, IQR = 2.88–3.92) (*p* = 0.019), as well as compared to those with 16–20 years of experience (median = 3.6, IQR = 2.84–4.12) (*p* = 0.041). In turn, physicians with 16–20 years of experience registered a higher score than those with 26–30 years of experience (median = 2.38, IQR = 2.12–3.24) (*p* = 0.041), indicating that, in this particular sample, the group of physicians with 16–20 years of experience was generally more reluctant to embrace digital technology.

A particularly interesting aspect of the study, which emerged from the data collected and analyzed, shows that dentists with experience between 0 and 3 years and those with experience between 16 and 20 years experience significantly greater difficulties in adapting to digital workflows. The less experienced group, despite having benefited from a more extensive digital education in college, faces challenges in applying theoretical knowledge in real clinical practice, facing a steep learning curve and limited practical support. On the other hand, the group with 16–20 years of experience received little or no digital training in the early years of their careers and developed solid analog workflows that were difficult to replace mid-career. Thus, this group may show greater resistance to adopting new systems. Compared to these two subgroups, dentists with 4 to 15 years of experience show lower levels of inadaptability, suggesting that they have benefited from progressive exposure to technology and frequent updates to their work infrastructure. The latter group, in turn, has benefited from digital knowledge acquired in academia and clinical practice with a strong digital character, requiring them to keep up with frequent updates to international protocols and recommendations. This dynamic highlights how both generational and professional factors contribute to adaptation difficulties. Younger dentists face practical barriers, while those mid-career face already ingrained methods.

Overall, these findings highlight the importance of tailored support, from hands-on mentoring for beginners to targeted retraining programs for mid-career clinicians, so that the integration of digital technology is as smooth as possible at all levels of experience. Overall, nuanced models of adaptation show that the success of implementing digital solutions depends on reducing both educational gaps and practical limitations. Correcting these divergences can create a unified practice environment in which all specialists capitalize on technological advances, leading to direct patient benefit and optimizing procedures for the real benefit of the physician by optimizing allocated resources, be they time, financial, or human resources involved.

### 3.9. Implications of AI in Dentistry

As dentistry moves firmly into the digital age, AI is beginning to influence the entire career path of the clinician, from pre-clinical training to decisions made at the dental chair, and its long-term value will depend on the extent to which these tools reinforce, rather than replace, the human dimensions of the medical act. In education, the virtual reality-based curriculum evaluated by Sukotjo and collaborators shows that immersive simulation not only accelerates the acquisition of procedural competence but also personalizes learning through real-time feedback, anticipating an AI-supported pedagogy in which algorithms tailor scenarios to each student’s performance and democratize access for those in remote or resource-limited areas [23].

Once in clinical practice, the high fidelity reported for modern intraoral scanners by Canullo and colleagues provides the quality data needed by systems relying on AI algorithms to detect early lesions, optimize implant positioning, and automate CAD/CAM flows with a consistency that surpasses manual interpretation [24]. However, technological precision is not enough: large epidemiologic studies consistently show that patients’ willingness to accept even painful treatments is influenced more by perceived trust and empathy than by the clinician’s technical competence. If algorithms are perceived as distancing the clinician, excessive digitization may erode this essential trust, leading to avoidance and increased anxiety. Therefore, the strategic goal should be augmentation, not replacement: AI should take over repetitive analytic tasks, freeing the clinician to communicate, reassure, and make decisions with the patient. Within this framework, AI has the potential to improve both clinical outcomes and the quality of the relationship that defines successful dental care [25].

The importance of the issue analyzed in this study is supported by a number of studies identified in the literature. We identified analyses of certain indicators that we have examined. Referring to the specialized literature and findings from other studies focused on diagnostic procedures in dentistry, a study conducted in 2025 on the capacity of AI-based software to diagnose periodontal diseases showed that the detection capabilities of the presented software are comparable to those of a specialized dentist with extensive experience in the field [26].

Also, studies show genuine benefits from implementing AI-based digital tools during the treatment planning stage. For example, by minimizing errors, improving workflow efficiency, and refining diagnostic precision, virtual articulators significantly enhance prosthodontic, orthodontic, and orthognathic treatments. This advanced technology improves condylar guidance accuracy and supports more reliable treatment modalities [27].

An interesting study conducted by the University of Trieste in Italy further substantiates the concept of involving AI in patient monitoring. Dentists are advised to take this approach into consideration by directing patients toward reliable sources of information. This research specifically examines the responses provided by ChatGPT version 3.5 to patients, as evaluated by specialists in the field, and the results are remarkable. The accuracy of the answers is notably high, indicating that virtual assistants are playing an increasingly significant role in patient education [28].

At the same time, we identified a set of intriguing findings from a study conducted in Germany (Eschert, 2022), where 23.2% of the surveyed dental practitioners evaluated their knowledge regarding digital technologies based on AI-driven tools as very poor, 37.1% assessed their knowledge as average, and 22.2% considered it above average [29].

Enhanced digital literacy through continuing education and hands-on training builds confidence and competence. Structured mentorships, pairing tech-competent mentors with colleagues less familiar with digital workflows, can further accelerate skill acquisition—an approach already proven to foster technology uptake in healthcare. Likewise, peer support networks like online forums, Facebook groups, or WhatsApp conversation groups facilitate knowledge-sharing and reduce professional isolation in remote areas [30]. By prioritizing individual and clinic-level skill development, rural practitioners can overcome adoption challenges, close the digital gap, and unlock the benefits of modern dental technology, including greater efficiency, improved diagnostic accuracy, and more equitable patient outcomes [31].

**Figure 8:**
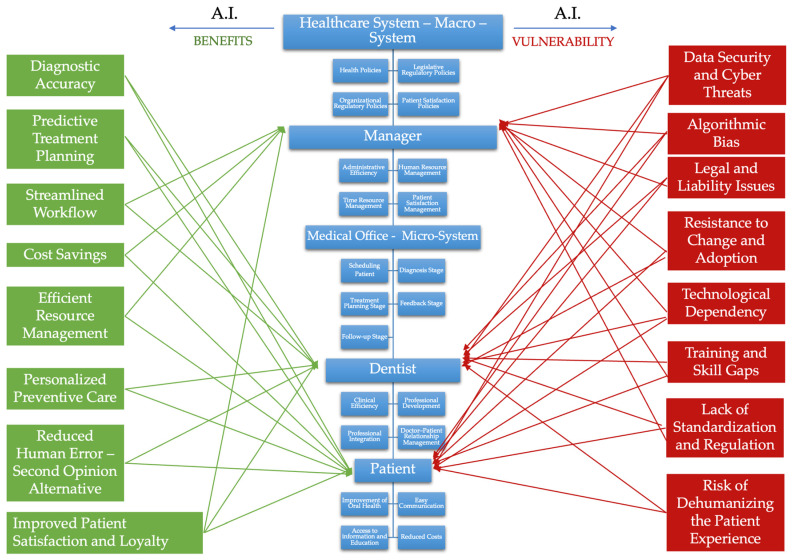
Illustrating dental health system and AI benefits and vulnerability.

For a better understanding of the whole process, we designed a diagram, as shown in Figure 8, that articulates a dual-facet framework that situates AI within the multilevel architecture of oral healthcare, contrasting its potential benefits with its attendant vulnerabilities. At the apex, the healthcare macro-system—defined by health, legislative, organizational, and patient-satisfaction policies—sets the regulatory perimeter inside which AI operates [32,33]. Beneath it, the managerial stratum mediates administrative efficiency, human-resource governance, temporal resource allocation, and patient-satisfaction management, while the medical office micro-system orchestrates the clinical journey from scheduling and diagnosis through treatment planning, feedback, and follow-up [34,35]. The dentist, responsible for clinical efficiency, professional development, and the doctor–patient relationship, interfaces directly with the patient, whose interests center on oral health improvement, ease of communication, informational access, and cost reduction [36].

On the left, eight AI-enabled advantages cascade across these tiers. Enhanced diagnostic accuracy and predictive treatment planning inform evidence-based decision-making at both managerial and clinical nodes [34]. Streamlined workflows, cost savings, and efficient resource management bolster macro- and meso-level organizational performance [37,38]. For the dentist–patient relationship, AI promises personalized preventive care, a reduction in human error through second-opinion support, and ultimately, improved patient satisfaction and loyalty [39,40]. These gains are visually connected by green vectors, underscoring their systemic diffusion.

Conversely, the right flank enumerates eight red-coded vulnerabilities that threaten the same levels. Data security and cyber threats imperil the integrity of patient records, while algorithmic bias jeopardizes diagnostic equity. Legal and liability issues, compounded by a lack of standardization and regulation, expose providers and managers to compliance uncertainty. Organizational inertia manifests as resistance to change and adoption, whereas technological dependency raises concerns about operational resilience [41,42]. At the human-capital level, training and skill gaps hinder effective implementation, and an excessive focus on automation risks dehumanizing the patient experience. The red arrows converging on managerial and clinical nodes illustrate how these hazards propagate through governance, practice, and patient care.

Understanding and strategically balancing these interlinked forces is indispensable for achieving ethically grounded, high-quality, and sustainable AI integration in dentistry.

### 3.10. The Importance of the Learning Curve

In relation to the learning curve, at this stage of implementation and integration of AI in medical practice, every dentist experiences a learning curve when beginning to utilize AI-based tools, and understanding this curve—particularly its initial stages—is critical for effective innovation implementation. The classic model of the four stages of competence (also known as the “conscious competence model”) describes progression from unconscious incompetence (when individuals do not know what they do not know) to conscious incompetence (when they recognize their knowledge gaps and begin learning), then to conscious competence (when they can perform the new skill but only with deliberate effort), and ultimately to unconscious competence (mastering the skill effortlessly, automatically).

In the context of dentists’ adoption of AI, the initial stage—unconscious incompetence—holds particular significance [42,43]. In this phase, the dentist neither understands nor knows how to effectively use AI, nor does he or she acknowledge these knowledge deficits, often denying the usefulness of the new skill. In other words, they are unaware of their ignorance. Many experienced dentists, trained during a period when AI was absent from medical practice, may initially be unaware of the potential these tools offer and the necessity of learning them. They might mistakenly believe their practice is already optimal without AI, dismissing AI as a passing trend. This attitude is frequently coupled with overconfidence in traditional methods and an underestimation of the difficulty of correctly using the technology. Unfortunately, this mindset may lead to passive resistance: dentists may not actively pursue further training, thus falling behind colleagues who are progressing. Moreover, the lack of awareness about their incompetence can lead to errors—for instance, a dentist might superficially adopt AI software without adequate training, subsequently placing unwarranted trust in its results, potentially resulting in clinical mistakes. This represents the paradox of unconscious incompetence: significant risks exist, but individuals fail to recognize them and thus do not take necessary precautions. All these genuine risks, coupled with the dentist’s inability to perceive them, may foster defensive practice behaviors [44,45]. As a consequence, the dentist may either become overly reliant on AI recommendations to justify every clinical decision or, conversely, reject modern systems entirely. The primary risk for the dentist in both cases is the erosion of clinical autonomy and potential exposure to legal accountability for diagnostic or therapeutic errors induced by AI. Concurrently, the patient might suffer from receiving excessive or unnecessary treatments resulting from overly rigid or excessively cautious interpretations of AI recommendations, negatively impacting both the quality of care and trust in their relationship with the dentist [46,47].

To facilitate AI adoption, it is crucial to accelerate dentists’ progression from this initial stage toward conscious incompetence—essentially, helping them recognize what they do not know and the value of acquiring this knowledge. This can be accomplished through education and clearly presenting both the benefits and vulnerabilities of AI via demonstration workshops, introductory courses on AI in dentistry, consultation of specialized research studies, and, importantly, learning from the experiences and mistakes of others. When dentists practically observe how an algorithm can detect hidden lesions or save time in treatment planning, they may experience a revelatory moment (“Eureka!”) [48] that moves them into the stage of conscious incompetence—recognizing their need for further learning. This is when the motivation to learn arises, although it may initially be accompanied by frustration at realizing the many stages of catching up needed, or anxiety about being left behind. Educators and opinion leaders in the medical field play a critical role in transforming this frustration into curiosity and providing positive guidance.

The results of this study reveal a profound need for change through learning, a difficult process of adaptation and acceptance of the cycle of learning, unlearning, and relearning, which is essential in this changing world. With this in mind, we consider it essential to implement a dual perspective in terms of European policies and strategies with national implementation. On the one hand, we discussed the need to ensure a high level of safety for practitioners in the use of AI by developing the legal framework for regulating its intervention in healthcare. On the other hand, we discussed the need to fill the information gaps of both students who will be future doctors and doctors whose learning process took place before AI was introduced into professional practice. Thus, strategies should aim to adapt the university curriculum to include learning elements specific to the digital age for students, as well as organizing certifications for the acquisition of skills by doctors already in the workforce. To ensure that the full potential of AI is realized in dentistry, it is imperative to construct a regulatory architecture that upholds ethical standards, clinical safety, and sustainability. Achieving this goal requires coordinated efforts among regulatory bodies, academic institutions, and professional organizations, ensuring that the principles of responsibility, transparency, and patient-centered care guide implementation. In this light, AI must not be perceived as a replacement for clinical expertise, but rather as a complementary instrument that augments professional judgement, reinforces medical precision, and humanizes clinical interactions in the digital era.

This study, conducted by the research team, has certain limitations regarding the number of participants, the restricted environment of their practical activity, the research being conducted only in Bucharest and adjacent areas, and also the extremely rapid dynamics of the field analyzed. It is necessary that in future, based on the results obtained and the conclusions drawn from this research, the field of AI and its integration in the current practice of dentists should include as many adjacent studies as possible. In order to better understand the world in which dentists work, we also need to address the challenges of the future.

## 4. Conclusions

The integration of AI into dental medicine represents a critical catalyst for the transformation of the doctor–patient dynamic. By facilitating the optimization of both clinical and administrative processes and enabling a more equitable distribution of resources, AI contributes significantly to reducing disparities in access to oral healthcare. Moreover, its capacity to enhance communication, enable continuous treatment monitoring, and improve diagnostic precision supports a more personalized and effective dispensary process. These advancements collectively foster stronger patient loyalty, elevate satisfaction levels, and promote the organic development of dental practices through enhanced trust and engagement. Change is no longer imminent; it is already unfolding. It falls upon each of us to embrace it, comprehend its implications, and utilize its potential to the highest degree. In conclusion, Marcus Tullius Cicero’s enduring relevance serves as a guiding principle: *“Character without intelligence can accomplish much; intelligence without character can accomplish nothing”*.

## Figures and Tables

**Table 1:** Tabular representation of perceived difficulty by age group (* Fisher’s Exact Test).

Age/ Difficulty	Low		Moderate		Raised		*p* *
	Nr.	%	Nr.	%	Nr.	%	
**25–30 years**	35	29.7%	15	18.3%	7	35%	<0.001
**31–35 years**	**48**	**40.7%**	**17**	**20.7%**	**1**	**5%**	
**36–40 years**	18	15.3%	18	22%	0	0%	
**41–45 years**	11	9.3%	14	17.1%	1	5%	
**46–50 years**	**4**	**3.4%**	9	11%	**4**	**20%**	
**51–55 years**	**0**	**0%**	**4**	**4.9%**	**3**	**15%**	
**56–60 years**	**2**	**1.7%**	5	6.1%	**3**	**15%**	
**>60 years**	**0**	**0%**	0	0%	**1**	**5%**	

**Table 2:** Tabular representation of perceived difficulty in relation to professional experience (* Fisher’s Exact Test).

Experience/ Difficulty	Low		Moderate		Raised		*p* *
	Nr.	%	Nr.	%	Nr.	%	
**0–3 years**	35	29.7%	16	19.5%	7	35%	<0.001
**4–10 years**	**57**	**48.3%**	**23**	**28%**	**1**	**5%**	
**11–15 years**	16	13.6%	23	28%	0	0%	
**16–20 years**	7	5.9%	11	13.4%	4	20%	
**21–25 years**	**1**	**0.8%**	4	4.9%	**3**	**15%**	
**26–30 years**	**2**	**1.7%**	4	4.9%	**4**	**20%**	
**31–35 years**	**0**	**0%**	1	1.2%	**1**	**5%**	

**Table 3:** Tabular representation of willingness by age group (* Fisher’s Exact Test).

Age/ Willingness	Low		Moderate		Raised		*p* *
	Nr.	%	Nr.	%	Nr.	%	
**25–30 years**	3	12%	12	18.2%	42	32.6%	<0.001
**31–35 years**	4	16%	**12**	**18.2%**	**50**	**38.8%**	
**36–40 years**	4	16%	13	19.7%	19	14.7%	
**41–45 years**	3	12%	**13**	**19.7%**	**10**	**7.8%**	
**46–50 years**	7	28%	8	12.1%	2	1.6%	
**51–55 years**	2	8%	3	4.5%	2	1.6%	
**56–60 years**	1	4%	5	7.6%	4	3.1%	
**>60 years**	1	4%	0	0%	0	0%	

**Table 4:** Tabular representation of willingness in relation to professional experience (* Fisher’s Exact Test).

Experience/ Willingness	Low		Moderate		Raised		*p* *
	Nr.	%	Nr.	%	Nr.	%	
**0–3 years**	3	12%	12	18.2%	43	33.3%	<0.001
**4–10 years**	6	24%	**16**	**24.2%**	**59**	**45.7%**	
**11–15 years**	4	16%	**19**	**28.8%**	**16**	**12.4%**	
**16–20 years**	**8**	**32%**	**10**	**15.2%**	**4**	**3.1%**	
**21–25 years**	1	4%	4	6.1%	3	2.3%	
**26–30 years**	2	8%	4	6.1%	4	3.1%	
**31–35 years**	1	4%	1	1.5%	0	0%	

**Table 5:** A tabular representation of the use of digital tools by age group (* Fisher’s Exact Test).

Age/Utilization	Absent		Present		*p* *
	Nr.	%	Nr.	%	
**25–30 years**	10	23.3%	47	26.6%	0.003
**31–35 years**	**7**	**16.3%**	**59**	**33.3%**	
**36–40 years**	5	11.6%	31	17.5%	
**41–45 years**	6	14%	20	11.3%	
**46–50 years**	**10**	**23.3%**	**7**	**4%**	
**51–55 years**	2	4.7%	5	2.8%	
**56–60 years**	3	7%	7	4%	
**>60 years**	0	0%	1	0.6%	

**Table 6:** A tabular representation of the distribution of practitioners according to their level of experience and the use of digital technology (* Fisher’s Exact Test).

Experience/Utilization	Absent		Present		*p* *
	Nr.	%	Nr.	%	
**0–3 years**	10	23.3%	48	27.1%	0.006
**4–10 years**	**8**	**18.6%**	**73**	**41.2%**	
**11–15 years**	9	20.9%	30	16.9%	
**16–20 years**	**10**	**23.3%**	**12**	**6.8%**	
**21–25 years**	2	4.7%	6	3.4%	
**26–30 years**	4	9.3%	6	3.4%	
**31–35 years**	0	0%	2	1.1%	

## Data Availability

The original contributions presented in this study are included in the article. Further inquiries can be directed to the corresponding author.

## Supplementary Materials

The following supporting information can be downloaded at: https://www.mdpi.com/article/10.3390/dj13090390/s1, Document S1: Questionnaire for Dentists
