# Supplementary material for: Embracing Artificial Intelligence in Dental Practice: An Exploratory Study of Romanian Clinicians’ Perspectives and Experiences
Source: Dent J (Basel). 2025 Aug 27;13(9):390. doi: 10.3390/dj13090390 (PMC12468662; doi:10.3390/dj13090390)
Supplement: Supplementary file 1 [file dentistry-13-00390-s001.zip › dentistry-3787643-supplementary.pdf]

QUESTIONNAIRE FOR DENTISTS

1. What is your age:

   - Age: ____ ____

2. What is your gender:

   - Male
   - Female

3. Where do you currently work:
   - Rural
   - Urban

4. Professional qualification:
   - Dentist
   - Resident Physician
   - Specialist
   - Head Physician

5. Level of professional experience in providing specialized medical care:
   Example: (5 years)

   - ____ years

6. How would you rate the level of difficulty you encounter when using digital tools and information technology?

Low	Moderate	High
O	O	O

7. How would you rate your willingness to adopt new technologies based on artificial intelligence in your dental practice?

Low	Moderate	High
O	O	O

8. Do you currently use digital tools to facilitate your relationship with patients, medical staff, and your colleagues? (e.g., scheduling software, data transfer software, e-learning platforms, etc.)

   - Yes
   - No

Please rank each of the following questions on a scale of 1 to 5. For each question, you can only choose one of the five options.

- ***1 represents a minimal level of interest***
- ***2 representing a low level of interest***
- ***3 representing a moderate level of interest***
- ***4 representing a high degree of interest***
- ***5 representing a very high level of interest in the statement***

9. ***Identify the biggest challenges of the current patient scheduling system in the clinic where you work***

- Managing adequate time for each patient according to the treatment required, without disrupting the work schedule
- Obtaining the informed consent of the patient, following the regulations in force and completing the necessary medical documentation
- Ensuring a flexible schedule for dental emergencies without significantly affecting scheduled appointments
- Maintaining effective communication with the patient during the allotted time strengthens the doctor-patient relationship
- Comply with internal rules and regulations in the context of a dynamic schedule and varied treatments

10. ***Identify opportunities to improve the current appointment management process through digitisation and integration of artificial intelligence into current practice***

- Automatically optimise treatment intervals based on patient history and the average time required for the targeted intervention
- Integration of scheduling software with other electronic systems in the healthcare sector (electronic patient records, billing, etc.)
- Analysis based on information provided by the patient prior to their visit to the practice through a remote assessment and allocation of the necessary time slot in the scheduler
- Increased patient access to personal medical information and the possibility of personalising the method of communication with the attending physician
- Up-to-date updates of all digital platforms related to current practice, including those related to legislative regulations, communication with the National Health Insurance House, and storage of sensitive information.

11. ***Identify potential threats posed by the digitisation and integration of artificial intelligence to your patient scheduling process***

- Rapid technological advances, with scheduling systems become obsolete in a relatively short time, requiring costly updates or complete replacement.
- Digital data storage and its use by various third parties increases the risk of a security breach, exposing sensitive patient information.
- The algorithms used in automatic scheduling can have errors that lead to scheduling conflicts or unproductive gaps.
- Creation of a dependency of the staff involved on automation for patient interaction, leading to a deterioration of the doctor-patient relationship in the medium and long term.
- Damage to the doctor-patient relationship through depersonalization of communication with the patient

12. Identify the biggest challenges you face in the diagnosis phase of your patients

- Lack of access to modern diagnostic techniques and limited capacity to perform paraclinical investigations due to limited resources or high costs
- Establishing a definitive dental diagnosis correlated with the patient's general health and related conditions
- Promoting and respecting the right to a second medical opinion in the context of personal reputational risk
- Lack of access to electronic patient records to monitor long-term medical developments
- Obtaining complex presumptive diagnoses that include all of the patient's symptoms

13. Identify opportunities to improve the current diagnostic process through digitisation and integration of artificial intelligence into current practice

- Digitising diagnostic processes, speeding up the process of obtaining results and reducing the time needed to interpret them
- Compiling the diagnoses obtained and summarising the presumptive diagnoses, providing personalised assessments, increasing the accuracy of the diagnosis, and reducing the possibility of human error
- Integrating diagnostic software that uses artificial intelligence with smart devices that measure vital parameters in everyday life to identify certain risk factors related to oral health and overall health
- Digitising diagnostic processes that open up opportunities for the development of telemedicine and increase patient access to specialised health services and the right to a second opinion
- Transforming specialist medical information issued by a specialist into information that is easy to understand for the general public (AI-generated images, three-dimensional models, etc.)

14. Identify potential threats posed by the digitisation and integration of intelligence in the diagnosis stage

- Risk of misdiagnosis by calibrating the sensitivity of interpretation algorithms too high, thus detecting normal variations as pathological, or calibrating the sensitivity too low, with the risk of overlooking obvious symptoms
- Dependence of medical staff on algorithms involved in artificial diagnosis, leading to a lack of training in conventional diagnosis and neglect of established clinical skills
- The digital collection and storage of diagnostic data increases vulnerability to cyber attacks aimed at stealing sensitive medical information
- High initial investment and maintenance costs, and dependence on an external technology provider
- Negative impact of technology on the doctor-patient relationship with people who are reluctant to adopt new diagnostic methods and new technologies

15. Identify the biggest challenges that arise when establishing a treatment plan for your patients

- Setting realistic expectations and outcomes, respecting competence limits and applicable legislation
- Fully presenting the Informed Patient Agreement to the patient and providing detailed information about the benefits, risks involved, and viable alternatives to the proposed treatment plan
- Compliance with the internal rules and regulations of the healthcare facilities where you work to avoid any interference with the treatment plan
- Respecting and encouraging the right to a second medical opinion, with the risk of refusing the proposed treatment plan
- Carrying out treatments that take into account personal or religious beliefs

16. Identify opportunities to improve the current treatment plan development process through digitisation and integration of artificial intelligence into current practice

- Implement artificial intelligence-based applications that improve the accuracy of radiological diagnosis and generate treatment suggestions
- Customisation of treatment plans initiated by the physician, including the patient's financial and time resources in the calculation algorithms.
- Complex analysis of the impact of implementing a specific oral and dental treatment plan on the patient's general condition and associated conditions
- Simulation of the results of the proposed treatment plan, including images of the patient at the end of the treatment plan
- Cost/benefit analysis of two or more proposed treatment plans

17. Identify possible threats posed by the processes of digitisation and integration of artificial intelligence in the treatment plan stage

- High acquisition, update, and maintenance costs, which may lead to an increase in the fees charged by the doctor to offset the costs
- Unauthorized access to sensitive patient treatment plan data and the possibility of unethical use of this data
- Generation of flawed treatment plans due to software errors and the presence of certain algorithmic biases that may affect the fairness of treatment, leading to possible allegations of discrimination
- Creation of a dependence on technology and loss of the doctor's clinical skills, as well as a reduction in the doctor's role in the decision-making process of the treatment plan
- Reduced personal interaction between the physician and the patient, which may lead to mistrust in the proposed therapeutic strategy

18. Identify the biggest challenges that arise during the feedback stage

- Patients are unwilling to provide feedback, and when they do, it is not always honest
- Feedback collection methods (written questionnaire, online, verbal feedback, etc.) are time-consuming and difficult to interpret
- Additional time, financial, and human resources are needed to implement the suggestions received
- Reluctance and resistance of staff involved in patient treatment to changes proposed through the feedback method
- Unrealistic or impractical suggestions proposed by patients

19. Identify opportunities to improve the current feedback process through digitisation and integration of artificial intelligence into current practice

- Quick and efficient collection of feedback, saving time for staff involved in patient treatment, as well as automatic analysis of responses received
- Quickly identify recurring issues, generating detailed reports on trends in patient responses
- Prioritize critical issues and focus on corrective and improvement actions
- Monitoring progress due to the implementation of suggestions received, evaluating their effectiveness and providing data on the benefits of their implementation
- Generating interactive reports for patients with the status of implementation of the feedback provided

20. Identify potential threats posed by digitisation and intelligence integration processes in the feedback stage.

- Errors in interpreting feedback and loss of human contact in interactions with patients, who may perceive the process as impersonal
- Unauthorized access to patient data and data provided by patients, and unethical use (sharing data without consent)
- Inadequate storage of sensitive data collected through the feedback process and the possibility of security breaches
- Excessive dependence on technology and damage to the doctor-patient relationship
- Suggesting increased resource consumption (beyond the capabilities of medical staff) to implement the suggestions received

21. Identify the biggest challenges encountered during the follow-up stage

- Lack of an effective patient follow-up system and ineffective communication with patients
- Patients' misunderstanding of the importance of the follow-up process, followed by a deterioration in their health and an increase in the number of dental emergencies
- The complexity of implementing a follow-up program and resistance to implementation by staff involved in patient treatment
- High consumption of time, financial, and human resources for the implementation of a dispensary system

- Patient education and awareness. Many patients do not have sufficient knowledge about the importance of oral hygiene and regular visits to the dentist, which complicates the prevention process

22. Identify opportunities to improve the current dispensary (follow-up) process through digitisation and integration of artificial intelligence into current practice.

- Create and maintain an efficient dispensary system by automating appointments and reminders, ensuring constant communication with patients
- Educate patients by distributing personalized and interactive materials about the importance of dispensary care and oral hygiene
- Continuous monitoring of health status and the ability to detect problems at an early stage, reducing the risk of long-term complications
- Increasing access to follow-up methods through the use of telemedicine and remote consultation
- Compiling relevant statistics and analyses, followed by their graphical representation for the personalized promotion of dental prevention within the practice

23. Identify potential threats posed by digitisation and intelligence integration processes in the follow-up stage

- Creating an automated system for appointments and reminders could lead to excessive dependence on it. In the event of technical failures or cyber attacks, the practice's ability to manage appointments and communicate with patients could be severely affected, causing dissatisfaction among patients.
- Automation and the use of telemedicine for follow-up may reduce direct interaction between doctors and patients. This could lead to a decline in patient trust in medical staff, affecting the quality of the doctor-patient relationship.
- The use of artificial intelligence in health monitoring and early problem detection relies on algorithms that may be susceptible to errors or inaccuracies. False positive or false negative diagnoses may lead to unnecessary treatment or the overlooking of real conditions, compromising patient health.
- The distribution of personalized educational materials involves the collection and storage of large amounts of personal and medical data. There is a risk that this information could be accessed without authorization, compromising the confidentiality of sensitive patient data and exposing patients to possible data abuse.
- Implementing and maintaining artificial intelligence systems for dispensing medication can involve significant costs.
